# Feasibility of robot-based perturbed-balance training during treadmill walking in a high-functioning chronic stroke subject: a case-control study

**DOI:** 10.1186/s12984-018-0373-z

**Published:** 2018-04-11

**Authors:** Zlatko Matjačić, Matjaž Zadravec, Andrej Olenšek

**Affiliations:** University Rehabilitation Institute, Republic of Slovenia, Linhartova 51, SI-1000 Ljubljana, Slovenia

**Keywords:** Ankle strategy, Hip strategy, Perturbed walking, Stepping response, Center of mass, Center of pressure, Ground reaction forces

## Abstract

**Background:**

For stroke survivors, balance deficits that persist after the completion of the rehabilitation process lead to a significant risk of falls. We have recently developed a balance-assessment robot (BAR-TM) that enables assessment of balancing abilities during walking. The purpose of this study was to test feasibility of using the BAR-TM in an experimental perturbed-balance training program with a selected high-functioning stroke survivor.

**Methods:**

A control and an individual with right-side chronic hemiparesis post-stroke were studied. The individual post-stroke underwent thirty sessions of balance-perturbed training that involved walking on an instrumented treadmill while the BAR-TM delivered random pushes to the participant’s pelvis; these pushes were in various directions, at various speeds, and had various perturbation amplitudes. We assessed kinematics, kinetics, electromyography, and spatio-temporal responses to outward-directed perturbations of amplitude 60 N (before training) and 60 N and 90 N (after training) commencing on contact of either the nonparetic-left foot (LL-NP/L perturbation) or the paretic-right foot (RR-P/R perturbation) while the treadmill was running at a speed of 0.4 m/s.

**Results:**

Before training, the individual post-stroke primarily responded to LL-NP/L perturbations with an in-stance response on the non-paretic leg in a similar way to the control participant. After training, the individual post-stroke added adequate stepping by making a cross-step with the paretic leg that enabled successful rejection of the perturbation at lower and higher amplitudes. Before training, the individual post-stroke primarily responded to RR-P/R perturbations with fast cross-stepping using the left, non-paretic leg while in-stance response was entirely missing. After training, the stepping with the non-paretic leg was supplemented by partially recovered ability to exercise in-stance responses on the paretic leg and this enabled successful rejection of the perturbation at lower and higher amplitudes. The assessed kinematics, kinetics, electromyography, and spatio-temporal responses provided insight into the relative share of each balancing strategy that the selected individual post-stroke used to counteract LL-NP/L and RR-P/R perturbations before and after the training.

**Conclusions:**

The main finding of this case-control study is that robot-based perturbed-balance training may be a feasible approach. It resulted in an improvement the selected post-stroke participant’s ability to counteract outward-directed perturbations.

**Trial registration:**

ClinicalTrials.gov Identifier: NCT03285919 – retrospectively registered.

**Electronic supplementary material:**

The online version of this article (10.1186/s12984-018-0373-z) contains supplementary material, which is available to authorized users.

## Background

For community-dwelling stroke survivors, falls are common and mainly occur during walking [1]. Balance and gait deficits that persist after the completion of the rehabilitation process are significant risk factors and may not be detected by clinical scales as these do not evaluate a person’s ability to respond to unexpected perturbations during walking [2]. There is a particular risk of hip fracture from more frequent sideways falls [1]. These often result from inadequate capacity to respond efficiently to a loss of balance during standing and walking that occurs as a consequence of a floor slip or a push that acts in a frontal plane [3]. Stroke survivors generally have responses to external perturbations that are delayed and less coordinated than those of healthy individuals [4].

Previous studies investigating balancing responses following pushes in the frontal plane during walking have shown that healthy individuals use two strategies to counteract perturbations [5]. The first strategy is an in-place response: modulating the center of pressure (COP) under the in-stance leg (ankle strategy), possibly combined with a modulation of the horizontal component of ground reaction forces (GRF) (hip strategy) [6]. The second strategy is a stepping response: placing the swinging leg in a new location that facilitates an adequate dynamic relation of COP and center of mass (COM) combined with appropriate modulation of GRF to enable successful recovery from the perturbation [7]. Since the stepping strategy can only be applied over the next step(s), the location of the next foot placement(s) after a perturbation is a very important factor in the efficiency of perturbation rejection. If the foot placement in the first and subsequent steps following the perturbation is not adequate, there may be further self-induced perturbations and these may not be limited to the frontal plane but also occur in the sagittal plane [4, 8].

If an outward perturbation of sufficient amplitude is delivered when the foot of the non-impaired leg makes contact with the ground, employing a stepping strategy that requires adequate positioning of the impaired leg may be a considerable challenge for even high-functioning stroke survivors. This is because the cross-step may need to be executed with the impaired leg and, at the same time, collision with the non-impaired stance leg must be avoided. Similarly, an outward perturbation delivered when the foot of the impaired leg makes contact may also be difficult for a stroke survivor to cope with due to the diminished capabilities of the impaired leg to produce adequate in-stance response [3].

It has been postulated that if one wants to learn or acquire a specific movement skill one needs to practice that specific movement [9]. If we apply this to the balancing skills necessary to effectively counteract perturbations during walking, it would mean that high-functioning individuals post-stroke need to be exposed to realistic balance-threatening situations to enable development of efficient balancing skills within the limits of their specific gait/balance deficits. Verheyden et al. [10] reviewed interventions for preventing falls in people after stroke and found that exercises that aimed to improve mobility and balance had no effects in terms of reducing the number of falls. Mansfield et al. [11] have shown that when individuals post-stroke are walking and have their balance challenged by external pushes delivered manually by therapists, they improve their reactive balancing capabilities. This method leads to a reduced number of falls in comparison to traditional approaches to balance training.

We have recently developed a balance-assessment robot (BAR) [12]: an admittance-controlled haptic robot that interfaces with the pelvis of a walking subject. In combination with an instrumented treadmill (TM), this enables: (i) application of perturbing pushes to the pelvis, and (ii) assessment of the resulting balancing responses in terms of COM, COP and GRF. The utility of the BAR-TM may not be limited to assessment; it could also be used for training balancing responses to perturbations delivered as COM displacements in fall-safe conditions and thus, possibly, facilitate development of efficient balancing responses across the post-stroke population.

The primary purpose of this pilot study was to test the feasibility of using a BAR-TM robot in an experimental perturbed-balance training program with a selected community-dwelling, high-functioning stroke survivor. We present a detailed case report on the performance of the individual post-stroke before and after the experimental training. Our main goal was to explore whether such a training program is feasible and, further, whether it can bring about improvement in balancing skills following outward-directed perturbations and how they compare to those of a matched, neurologically intact subject.

## Methods

### Case description – Participants

One stroke survivor (six months post-stroke, with resulting right-sided hemiparesis), and one healthy height- and weight-matched participant participated in the study. The individual post-stroke was 53 years old, had suffered a hemorrhagic stroke in the area of the basal ganglia, and had, prior to the start of this study, completed two months of a rehabilitation program at our institute. After completing rehabilitation, the individual post-stroke who volunteered to participate in this study continued to be physically active and could be regarded as high-functioning, community-dwelling person [1]. He lives independently in the community, and his functional ambulation category (FAC) before this study was the maximal 5 [13]. The study was approved by the Slovenian National Ethics committee and both participants signed informed-consent forms.

### Experimental environment

**Fig. 1:**
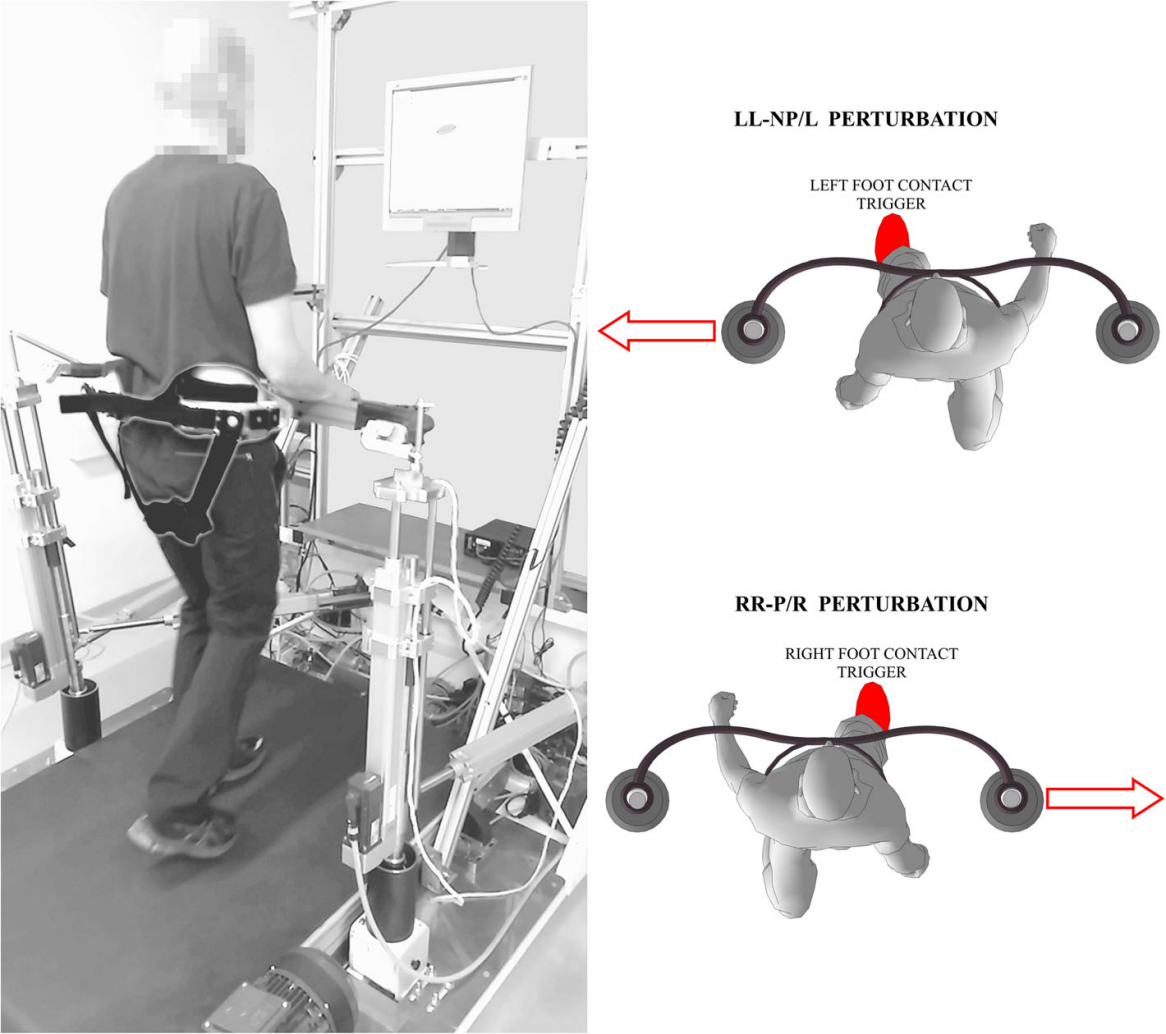
Photo of an individual post-stroke walking on an instrumented treadmill while being embraced by the BAR-TM perturbing device; computer screen shows the middle of the BAR-TM working space as well as the current position and orientation of the pelvis in transverse plane (left). Top view illustration of both outward perturbation directions: RR-P/R – perturbation to the right triggered at right-foot contact; LL-NP/L – perturbation to the left triggered at left-foot contact (right)

Figure 1 shows the experimental environment, which consisted of a balance-assessment robot and an instrumented treadmill (BAR-TM). The BAR-TM interfaces with the pelvis of a walking participant with six degrees of freedom (DOF). Five of the DOFs (translation of pelvis in sagittal, lateral, and vertical directions; pelvic rotation and pelvic list) are actuated and admittance-controlled, so providing transparent haptic interaction with negligible power transfer [8]. The sixth DOF (pelvic tilt) is passive. The BAR-TM is capable of delivering perturbations in the forward/backward and left/right directions but this study only considered outward perturbations in the frontal plane as depicted in Fig. 1. A detailed description of the BAR’s architecture, control, and performance is given in [12].

COM movement was estimated from the translational movement of the BAR-TM. It has been shown that such an approximation can be reliably applied during unperturbed walking [14] as well as during walking perturbed with moderate pushes applied to a pelvis [5]. The interaction forces between a walking participant and the BAR were assessed by force cells (K3D60a, ME Systeme GmbH). Records of the GRF and COP in the transversal plane during walking were obtained by means of four force transducers (K3D120, ME Systeme GmbH) placed underneath the treadmill according to the procedure described by Willems and Gosseye [15]. Spatio-temporal data were assessed by means of an Optitrack camera (NaturalPoint Inc.). Pasive reflective markers were placed on the participants’ feet (on the medial malleoli, and the first and fourth metatarsal joint) to create the kinematic model [12]. Sampling frequency for the kinematic and kinetic data was 50 Hz.

The electromyographic (EMG) activity of eight muscle groups (tibialis anterior – TA, soleus – SOL, gastrocnemius medius – GM, gastrocnemius lateralis – GL, rectus femoris – RF, semitendinosis – ST, gluteus medius – GMED and gluteus maximus – GMAX) was recorded bilaterally (using TelemyoMini 16, Neurodata, Vienna, Austria). The EMG electrodes were positioned over the palpated muscle bellies, the area underneath the electrodes was properly cleaned, and the electrical impedance was checked to assure optimal EMG recordings. The sampling frequency was set to 1 kHz. EMG signals were processed as follows: de-meaning of the raw signal, band-pass filtering (20–300 Hz), notch filtering (49–51 Hz), full-wave rectification, and moving-average window filtering (150 ms).

### Experimental training description

The individual post-stroke underwent thirty training sessions over a ten-week period with, on average, three training sessions taking place each week. Each training session started with 10–15 min of unperturbed treadmill walking. This initial warm-up period was followed by 30–45 min of perturbation training. The speed of the treadmill was between 0.3 m/s and 0.6 m/s. Perturbations were delivered approximately every six seconds in the forward, backward, left and right directions at one of two instants: at initial contact of the left foot or at initial contact of the right foot (Fig. 1). Perturbations took the form of a force of amplitude 50–100 N lasting for 150 ms [8, 12]. The time at which the perturbation commenced and the direction of the perturbation were block-randomized. The speed of the treadmill and the amplitude of perturbations for each training run (there were three runs of approximately ten minutes each per training session) was adjusted according to the subjective judgment of the researcher based on the standard deviations of kinematic and kinetic parameters assessed in previous training sessions. Once these standard deviations were considered to be sufficiently low an increment in either treadmill speed and /or amplitude of perturbations was proposed and implemented with the consent of the individual who was training. The speed and the amplitude were low in early sessions and were gradually increased as training progressed. In a single training session, each leg was subjected to at least twenty perturbations in each direction. Throughout all sessions, the pelvis of individual post-stroke was securely harnessed to the BAR-TM in a way that did not hinder movement but ensured safety (Fig. 1).

### Data assessment protocol

We completed two initial training sessions in order to experimentally determine the speed of the treadmill and perturbation amplitude that the individual post-stroke was comfortable with and that allowed him to produce repeatable responses. These values were 0.4 m/s and 60 N respectively (at higher perturbation amplitudes the participant was not able to withstand perturbations and was safely harnessed by BAR-TM mechanism). We then gathered data in an assessment session that we have termed as experimental condition BEFORE_60. After completion of the last training session, we repeated the assessment with the same parameters (experimental condition AFTER_60). Since, after the training, the individual post-stroke was able to comfortably negotiate higher perturbation amplitudes, we also assessed his performance at perturbation amplitude of 90 N (experimental condition AFTER_90). For the purposes of comparison of the acquired postural responses in the individual post-stroke, we also assessed postural responses in the control participant (experimental conditions CONTROL_60 and CONTROL_90). In all experimental conditions, the speed of walking was 0.4 m/s. Although we assessed postural responses to all of the randomly delivered perturbation directions, we here report only on the outward directions: LL-NP/L denotes perturbations that were directed to the left side as the left leg (in case of individual post-stroke the non-paretic leg) entered stance, while RR-P/R denotes perturbations that were directed to the right side as the right leg (in case of individual post-stroke the paretic leg) entered stance.

Additionally, the following battery of clinical outcome measures were used prior to and after the training period: Functional Ambulation Category (FAC) test, six-minute walking test (6-MWT), ten-meter walking test (10-MWT), timed Up&Go test (TUG) and four-step square test (FSST).

### Data processing

The COM, COP, GRF and EMG recordings were first segmented into strides with the gait cycle defined as the period between two consecutive left (for LL-NP/L responses) or right (for RR-P/R responses) heel strikes, as detected from COPx and COPy signals. Three full gait cycles, half of a cycle prior to and two and a half cycles after the onset of perturbation, were analyzed. Spatio-temporal responses were investigated in terms of step length, step width and step time where left (right) step length was taken to be the anterio-posterior distance between ankle markers at the moment of left (right) foot strike while left (right) step width was defined as the medio-lateral distance between the same markers. Step times were defined as the time elapsed between two consecutive left (right) and right (left) foot strikes. Step lengths, widths and times were measured across six steps (two steps prior to and four steps after perturbation commencement). For each experimental condition, EMG recordings across the three gait cycles considered were normalized to the maximal values for each muscle during unperturbed walking.

In each experimental condition, for perturbed walking, all trajectories were averaged across twenty repetitions of postural response; for the unperturbed walking, averages were taken across twenty blocks of three consecutive strides in each experimental condition. Step lengths, widths, and times were averaged for each of the six steps considered for each experimental condition.

## Results

### Responses following LL-NP/L outward perturbations

#### Kinematics and kinetics

**Fig. 2:**
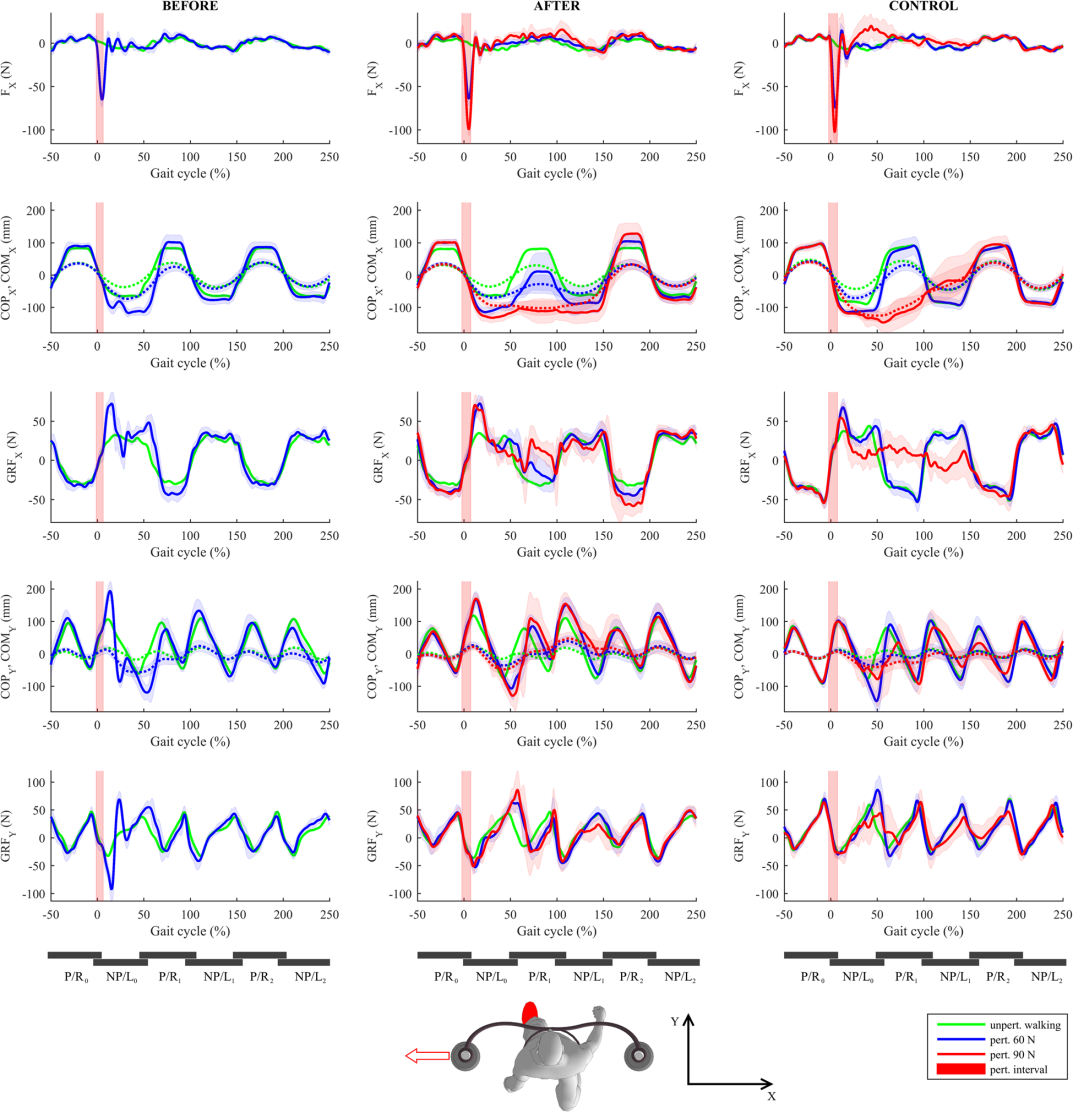
Kinematics and kinetics of balancing responses following LL-NP/L perturbation. The first row shows interaction forces between BAR-TM and participant’s pelvis in the frontal plane. The second row shows the trajectories of COPx (solid lines) and COMx (dotted lines), while the third row shows GRFx trajectories. The fourth row shows COPy (solid lines) and COMy (dotted lines) trajectories, and the fifth row shows GRFy trajectories. The left column shows the balancing responses of the individual post-stroke prior to the training (experimental condition BEFORE_60 – blue line) along with the unperturbed walking trajectories (green line). The middle column shows balancing responses of the individual post-stroke after the training (experimental conditions AFTER_60 – blue line and AFTER_90 – red line) along with the unperturbed walking trajectories (green line). The right column shows balancing responses of the control participant (experimental conditions CONTROL_60 – blue line and CONTROL_90 – red line) along with the unperturbed walking trajectories (green line). Half a stride prior to and two and a half strides following the perturbation commencement are shown. Stride is defined as the period between two consecutive left-foot contacts. The trajectories displayed show mean values and standard deviations of twenty balancing responses while, for clarity, only the mean values of twenty blocks of three consecutive strides of unperturbed walking are shown. Underneath each column, six consecutive stance phases of both legs are schematically indicated to enable easier cross-referencing with other figures. Subscript 0 denotes stance phases of both legs for steps taken before commencement of a perturbation, while subscripts 1 and 2 denote stance phases of each leg for steps taken after the commencement of a perturbation. P/R – paretic/right; NP/L – non-paretic/left

Figure 2 shows COP, COM, and GRF responses under the experimental conditions before training (BEFORE_60 – left column) and after training (AFTER_60 and AFTER_90 – middle column) compared to those of the control (CONTROL_60 and CONTROL_90 – right column).

Prior to the training (BEFORE_60), the individual post-stroke responded with an in-stance strategy on the non-paretic left leg (NP/L) by substantially shifting COPx laterally in the direction of the perturbation (0–25% of gait cycle – GC; Fig. 2, 2nd row); this was done partially through using an ankle strategy and partially by repositioning the stance foot by rapidly moving COPy (0–25% of gait cycle – GC; Fig. 2, 4th row) first toward the toes, to enable the heel to lift slightly and move laterally by pivoting the stance leg around the toes, followed by rapid displacement of COPy toward the heel to enable lifting slightly the forefoot, bringing it also laterally by pivoting the stance leg around the heel. In addition, a noticeable GRFx (Fig. 2, 3rd row) force impulse was generated under the non-paretic left leg (0–25% of GC) using the hip strategy. The in-stance response described fully arrested the movement of COMx (Fig. 2, 2nd row) initiated by the perturbation at about 25% of GC. However, the described displacements of COPy during the period from 0 to 50% of GC also provoked a substantial alternating movement of COMy (Fig. 2, 4th row) first backward (0–50% of GC) and later (100–150% of GC) forward. The in-stance postural response described was sufficient to efficiently counteract the perturbation and was similar to the control response (CONTROL_60) except for the in-stance repositioning of the foot.

After the training (AFTER_60), the individual post-stroke abandoned the strategy of repositioning the stance foot – once planted on the ground, the non-paretic left leg remained in the same location and orientation. This is confirmed by the shapes of COPy and GRFy (0 to 25% of GC, Fig. 2, 4th and 5th row) which are shape-wise similar to unperturbed walking and no longer display the oscillatory behavior noted prior to the training (BEFORE_60). Once again an in-stance strategy (0–50% of GC) composed of displacement of COPx (Fig. 2, 2nd row) laterally together with generation of a GRFx impulse (0–25% of GC; Fig. 2, 3rd row) on the non-paretic stance leg (NP/L0) was utilized. However, after the training, the stepping strategy was also partially utilized, as can be seen from the displacement of COPx (50–100% of GC), which differs noticeably from that in the unperturbed condition. The displacement of COMy backward (0–50% of GC) and then forward (100–150% of GC) was still present after training.

At the higher amplitude of perturbation (AFTER_90), the in-stance strategy alone was not sufficient. Apart from the ankle and hip in-stance strategies described, the first two steps use a stepping strategy, first with the right leg (P/R1) followed by the left leg (NP/L1) (50–150% of GC), which can be observed from COPx (50–150% of GC; Fig. 2, 3rd row). In the frontal plane, this postural response is very similar to the control response (CONTROL_90). In the sagittal plane, the responses are similar in the early phase (0–100%) but the individual post-stroke demonstrated a significant forward excursion of COMy (Fig. 2, 4th row) in the late phase (100–150%).

#### Spatio-temporal parameters

**Fig. 3:**
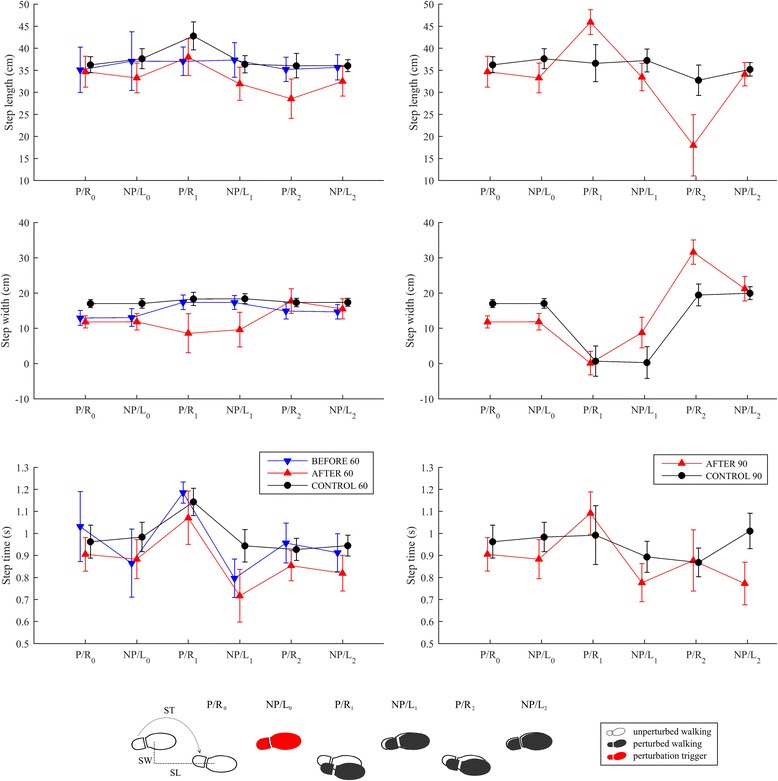
Mean values and standard deviations (twenty repetitions) of step lengths, widths, and step times for two steps prior to (P/R0 and NP/L0) and four consecutive steps following LL-NP/L perturbation commencement (P/R1, NP/L1, P/R2 and NP/L2). The left column shows data for experimental conditions BEFORE_60, AFTER_60 and CONTROL_60, while the right column shows data for experimental conditions AFTER_90 and CONTROL_90. Footprints illustrating unperturbed stepping and stepping following LL-NP/L perturbation with indication of step lengths (SL), step widths (SW) and step times (ST) is provided at the bottom for the experimental condition BEFORE_60

Figure 3 displays mean values and standard deviations for step lengths, widths, and times in all experimental conditions.

Step lengths were similar in the BEFORE_60, AFTER_60 and CONTROL_60 experimental conditions. There was a noticeable difference in step widths for the first two steps after the perturbation (P/R1 and NP/L1): substantially smaller step widths were recorded in the AFTER_60 condition than in the BEFORE_60 and CONTROL_60 conditions. It is evident from the temporal parameters for the BEFORE_60 and AFTER_60 experimental conditions that the step time of the first right step with the paretic leg (P/R) following the perturbation (P/R1) was much longer, facilitating the in-stance response on the left leg (NP/L0) described, which was similar to the control response (CONTROL_60). The second step of the left leg (NP/L1) that followed was substantially faster than the control response (CONTROL_60).

Comparison of experimental conditions AFTER_90 and CONTROL_90 shows a substantially longer first step (P/R1) for the individual post-stroke. The step widths and step times for the first step (P/R1) were similar, indicating the marked stepping response.

#### Electromyography

**Fig. 4:**
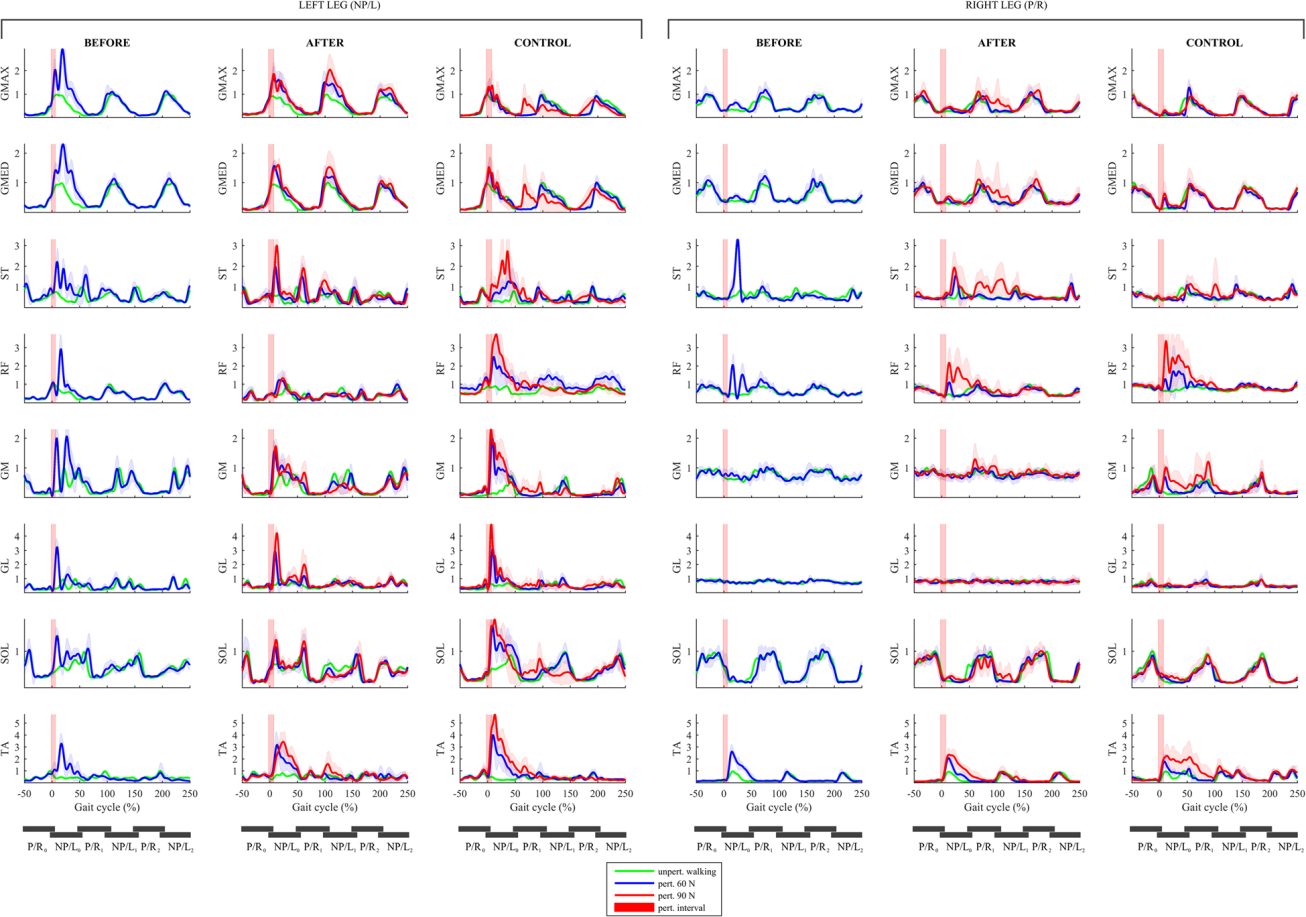
EMG responses following LL-NP/L perturbation. Left box shows responses for the muscles of the left leg (NP/L) while the right box shows responses for the muscles of the right leg (P/R). The left column in each box shows perturbed muscular activity of the individual post-stroke prior to the training (experimental condition BEFORE_60 – blue line) along with the unperturbed muscular activity (green line). The middle column in each box shows perturbed muscular activity of the individual post-stroke after the training (experimental conditions AFTER_60 – blue line and AFTER_90 – red line) along with the unperturbed muscular activity (green line). The right column in each box shows perturbed muscular activity of the control participant (experimental conditions CONTROL_60 – blue line and CONTROL_90 – red line) along with the unperturbed muscular activity (green line). Half of the stride prior to and two and a half strides following the perturbation commencement are shown. Stride is defined as the period between two consecutive left-foot contacts. The trajectories displayed show mean values and standard deviations of twenty balancing responses while, for clarity, only the mean values of twenty blocks of three consecutive strides of unperturbed walking are shown. Underneath each column, six consecutive stance phases of both legs are schematically indicated to enable easier cross-referencing with other figures

Figure 4 shows EMG responses of the lower limb muscles in both legs under all experimental conditions. A qualitative comparison of the muscular activity of the non-paretic left leg of the individual post-stroke and the left leg of the control participant shows substantial similarity in the action of all muscles throughout the response across all experimental conditions. In all experimental conditions, there is a marked increase of activity in all muscles from 0 to 50% of GC during perturbed walking when compared to unperturbed walking and this reflects the in-stance responses. There are however distinct differences predominantly in the SOL, GM, GL, RF and GMAX muscles of the non-paretic leg in the period from 0 to 50% of GC where oscillations related to re-positioning of the foot by pivoting first around the toes followed by pivoting around the heel can be clearly observed in the experimental condition BEFORE_60 while after the training this oscillatory behavior is no longer present in the experimental conditions AFTER_60 and AFTER_90. From 50 to 250% of GC, the muscular activity during perturbed and unperturbed walking is substantially similar. When comparing the muscular activity of the paretic leg of the individual post-stroke and the right leg of the control participant, a marked increase of activity in RF and TA from 0 to 50% of GC can be seen for all experimental conditions and a co-contraction of ST can be also observed in the individual post-stroke.

### Responses following RR-P/R outward perturbations

#### Kinematics and kinetics

**Fig. 5:**
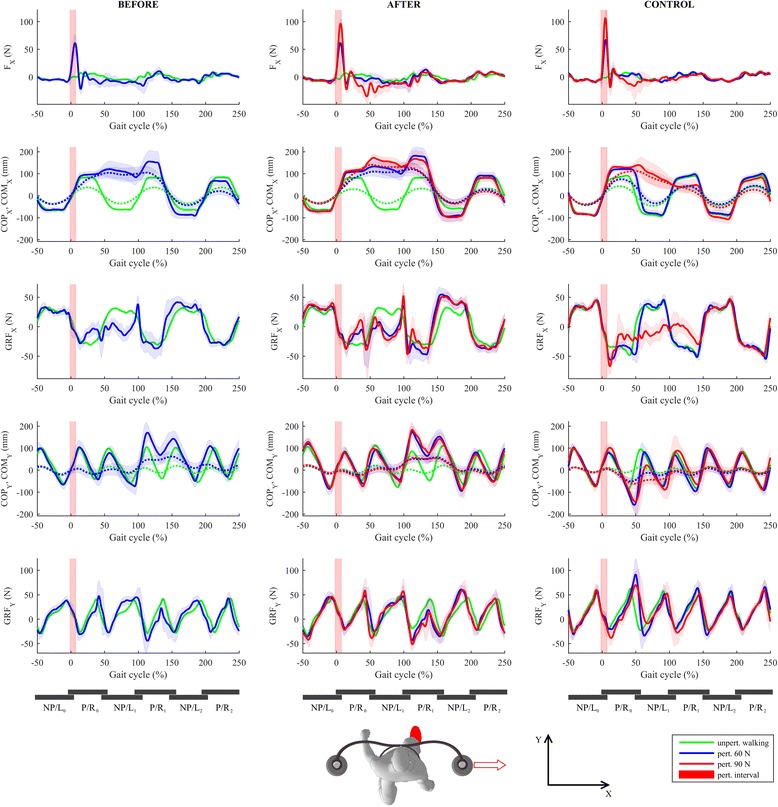
Kinematics and kinetics of balancing responses following RR_P/R perturbation. The first row shows interaction forces between BAR-TM and participant’s pelvis in the frontal plane. The second row shows the trajectories of COPx (solid lines) and COMx (dotted lines), while the third row shows GRFx trajectories. The fourth row shows COPy (solid lines) and COMy (dotted lines) trajectories, and the fifth row shows GRFy trajectories. The left column shows the balancing responses of the individual post-stroke prior to the training (experimental condition BEFORE_60 – blue line) along with the unperturbed walking trajectories (green line). The middle column shows balancing responses of the individual post-stroke after the training (experimental conditions AFTER_60 – blue line and AFTER_90 – red line) along with the unperturbed walking trajectories (green line). The right column shows balancing responses of the control participant (experimental conditions CONTROL_60 – blue line and CONTROL_90 – red line) along with the unperturbed walking trajectories (green line). Half a stride prior to and two and a half of strides following the perturbation commencement are shown. Stride is defined as the period between two consecutive right-foot contacts. Displayed trajectories show mean values and standard deviations of twenty balancing responses while, for clarity, only the mean values of twenty blocks of three consecutive strides of unperturbed walking are shown. Underneath each column, six consecutive stance phases of both legs are schematically indicated to enable easier cross-referencing with other figures. Subscript 0 denotes stance phases of both legs for steps taken before commencement of a perturbation, while subscripts 1 and 2 denote stance phases of each leg for steps taken after the commencement of a perturbation. P/R – paretic/right; NP/L – non-paretic/left

Figure 5 shows COP, COM, and GRF responses under the experimental conditions before training (BEFORE_60 – left column), after training (AFTER_60 and AFTER_90 – middle column) compared to those of the control (CONTROL_60 and CONTROL_90 – right column).

Prior to the training (BEFORE_60), an in-stance response is almost completely absent for the entire stance phase of the right-paretic leg. COPx after the perturbation (0–50% of GC; Fig. 5, 2nd row) is similar to that during unperturbed walking, indicating that an ankle strategy was not used, and the GRFx (Fig. 5, 3rd row) force impulse seen during the first 25% of the stance in response to a LL-NP/L perturbation is absent, indicating that a hip strategy is not used either. Therefore, throughout the entire stance phase of the impaired leg (0–50% of GC), COMx (Fig. 5, 2nd row) is almost freely displaced in the direction of the perturbation. Only after the non-paretic left leg (NP/L1) enters the stance (50–100% of GC) by making a cross-step – which can be seen from the substantial displacement of COPx (50–100% of GC) to the right when compared to unperturbed walking – is movement of COMx decelerated and then fully arrested by the next step of the paretic right leg when COPx is displaced even more to the right (P/R1; 100–150% of GC). The complete absence of in-stance response during P/R0 stance (0–50% of GC) also has implications for the movement of COMy (Fig. 5, 4th row). Throughout the first step with the left leg (NP/L1) following the perturbation, there is a pronounced rise in GRFy (50–100% of GC; Fig. 5, 5th row) compared to that seen during unperturbed walking and COMy (Fig. 5, 4th row) is thus accelerating forward. This self-induced forward perturbation was handled in the next step with the right leg (P/R1) by displacing COPy substantially forward (100–150% of GC) thus increasing GRFy (100–150% of GC; Fig. 5, 5th row) in the backward direction to decelerate COMy. This balancing strategy is very different to the control response (CONTROL_60) which was, to a large extent, a mirror image of the response to LL-NP/L perturbation.

**Fig. 6:**
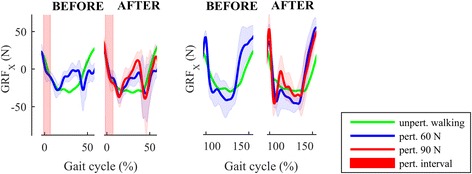
Comparison of GRFx responses following RR-P/R perturbation in two consecutive P/R steps before and after training in the individual post-stroke for all perturbing experimental conditions along with the unperturbed trajectories. After the training, hip-strategy GRFx force impulses immediately after right-foot contact can be seen for both perturbation strengths in both P/R0 (0–50% of GC; less pronounced) and P/R1 (100–150% of GC; more pronounced) stance phases

After the training (AFTER_60), the response remained qualitatively and functionally similar to that described above; however, there is a modest in-stance impulse in GRFx (0–50% of GC; Fig. 5, 3^nr^ row) followed by a bigger GRFx impulse in the second right leg (P/R1) stance phase (50–150% of GC), as indicated in Fig. 6.

At the higher amplitude of perturbation (AFTER_90), the response was similar to the one at smaller amplitude of perturbation (AFTER_60); however, the first step of the left leg was even more lateral as can be seen from COPx (NP/L1; 50–100% of GC; Fig. 5, 2nd row) due to the larger displacement of COMx produced by the stronger perturbation.

#### Spatio-temporal parameters

**Fig. 7:**
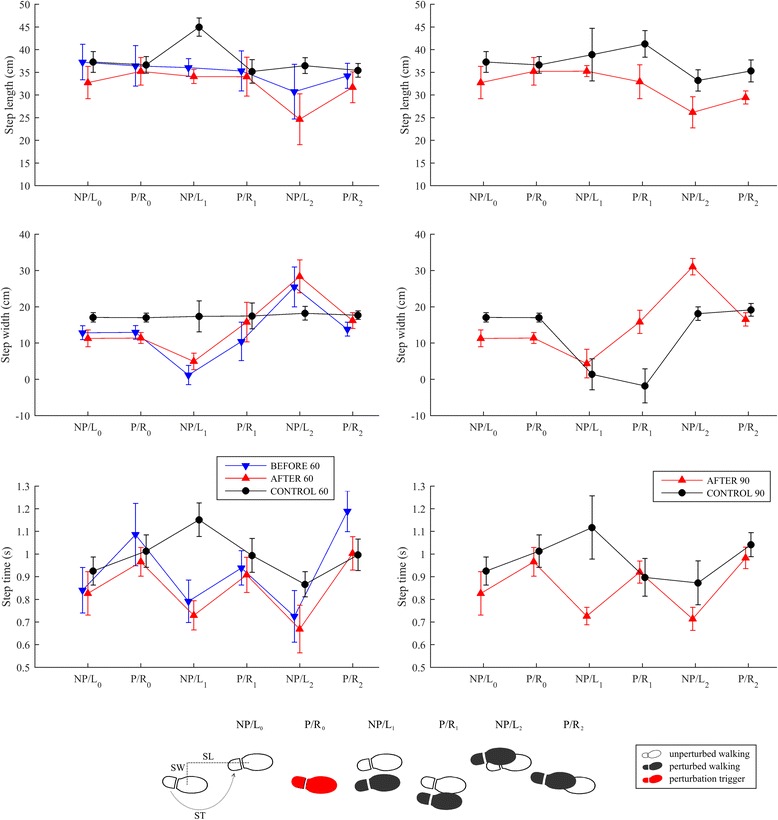
Mean values and standard deviations (twenty repetitions) of step lengths, step widths, and step times for two steps prior to (NP/L0 and P/R0) and four consecutive steps following RR-P/R perturbation commencement (NP/L1, P/R1, NP/L2 and P/R2). The left column shows data for experimental conditions BEFORE_60, AFTER_60 and CONTROL_60, while the right column shows data for experimental conditions AFTER_90 and CONTROL_90. Footprints illustrating unperturbed stepping and stepping following RR-P/R perturbation with indication of step lengths (SL), step widths (SW) and step times (ST) is provided at the bottom for the experimental condition BEFORE_60

Figure 7 displays mean values and standard deviations for step lengths, widths, and times in all experimental conditions.

Step lengths were similar in the BEFORE_60 and AFTER_60 experimental conditions, and differed markedly from the CONTROL_60 results, mainly in the first and the third steps after the perturbation. There was also a noticeable difference in step width for the first and third steps after the perturbation (NP/L1 and NP/L2): BEFORE_60 and AFTER_60 show a substantially smaller width for the first step and substantially bigger width for the third step than was seen in the CONTROL_60 experimental condition. It is evident from the temporal parameters for both the BEFORE_60 and AFTER_60 experimental conditions that the step time for the first step with the non-paretic left leg (NP/L1) following the perturbation was much shorter in the individual post-stroke. This enabled faster lateral displacement of the foot, and thus COPx, in the absence of an in-stance response.

A comparison of AFTER_90 and CONTROL_90 shows similar step lengths in each experimental condition but marked differences in step widths: the widths of the first steps (NP/L1) are similar but those of the next two steps (P/R1 and NP/L2) are substantially bigger for the individual post-stroke. Also the step time for the first step (NP/L1) after the perturbation is substantially shorter for the individual post-stroke and is similar to that seen in the experimental conditions BEFORE_60 and AFTER_60.

#### Electromyography

**Fig. 8:**
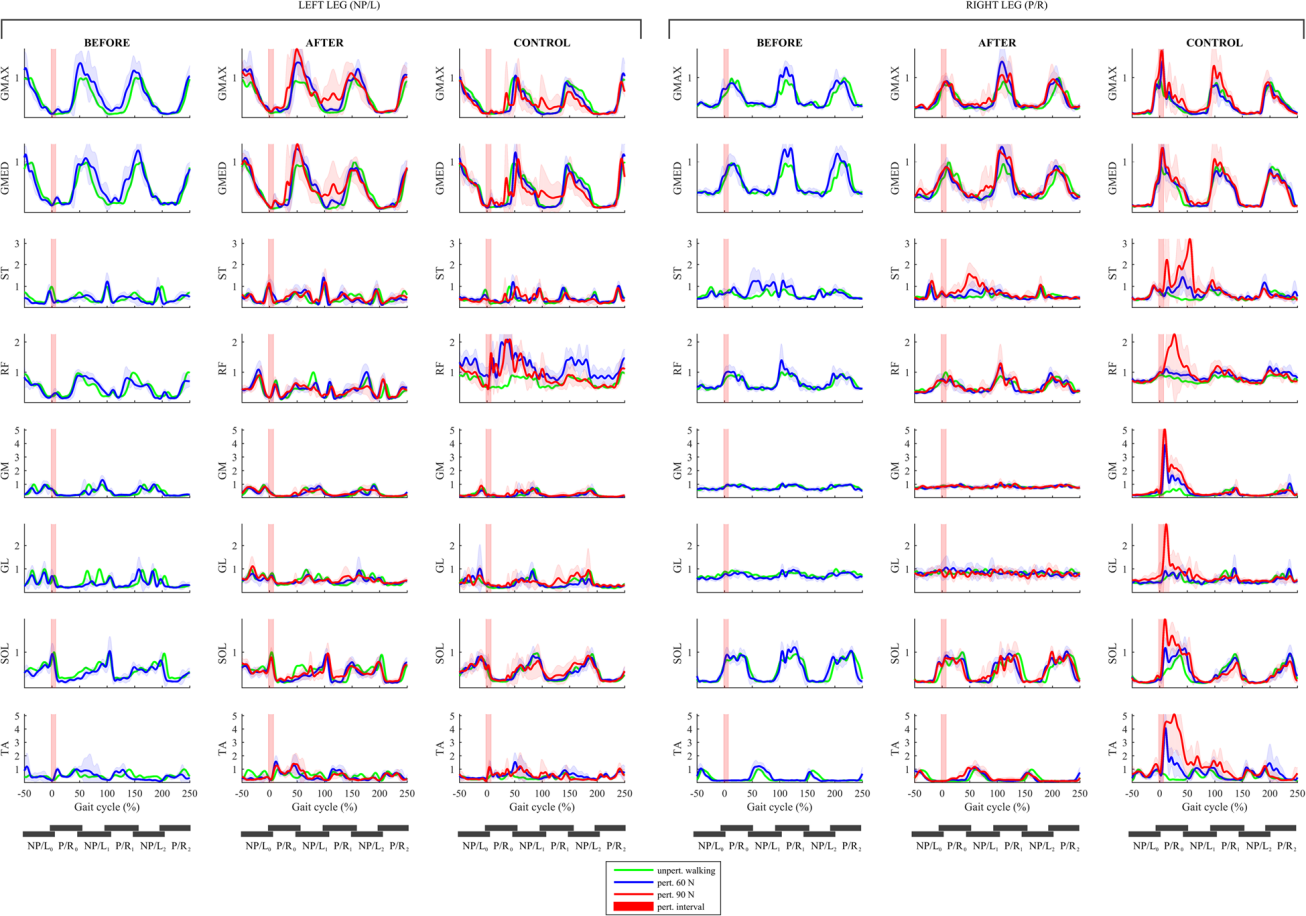
EMG responses following RR-P/R perturbation. Left box shows responses for the muscles of the left leg (NP/L) while the right box shows responses for the muscles of the right leg (P/R). The left column in each box shows perturbed muscular activity of the individual post-stroke prior to the training (experimental condition BEFORE_60 – blue line) along with the unperturbed muscular activity (green line). The middle column in each box shows perturbed muscular activity of the individual post-stroke after the training (experimental conditions AFTER_60 – blue line and AFTER_90 – red line) along with the unperturbed muscular activity (green line). The right column in each box shows perturbed muscular activity of the control participant (experimental conditions CONTROL_60 – blue line and CONTROL_90 – red line) along with the unperturbed muscular activity (green line). Half of the stride prior to and two and a half strides following the perturbation commencement are shown. Stride is defined as the period between two consecutive right-foot contacts. The trajectories displayed show mean values and standard deviations of twenty balancing responses while, for clarity, only the mean values of twenty blocks of three consecutive strides of unperturbed walking are shown. Underneath each column, six consecutive stance phases of both legs are schematically indicated to enable easier cross-referencing with other figures

Figure 8 shows EMG responses of lower limb muscles in both legs under all experimental conditions. A qualitative comparison of the muscular activity of the paretic leg of the individual post-stroke during unperturbed walking and perturbed walking shows substantially similar activation in all experimental conditions (BEFORE_60, AFTER_60 and AFTER_90) indicating an almost complete lack of the in-stance response displayed by the control participant through increased activation of all the muscles of the right leg, predominantly in the period from 0 to 50% of GC (experimental conditions CONTROL_60 and CONTROL_90). A qualitative comparison of the muscular activity of the non-paretic leg of the individual post-stroke and the left leg of the control participant showed there was substantially similar activation of all muscles in all experimental conditions.

#### Clinical outcome measures

Table 1 shows the performance of the individual post-stroke before and after the thirty training sessions and demonstrates improvement in all the assessed clinical outcome measures.

**Table 1:** Clinical outcome measures assessed in the post-stroke subject before and after the 30 training sessions

	Before training	After training
FAC	5/5	5/5
6MWT (m)	295	360
10-Meter walk test (s)	8.1	7.6
TUG (s)	12.3	10.6
FSST (s)	12.8	11.1

## Discussion

The main finding of this case-control study is that the experimental perturbed-balance training approach resulted in an improvement in the ability of the selected individual post-stroke to counteract outward-directed perturbations acting upon foot contact of either the non-paretic or the paretic leg. The primary evidence for this conclusion is the fact that, after training, the individual post-stroke was capable of successfully withstanding perturbations that were 50% stronger than those he was able to deal with prior to the training. The kinematics, kinetics, EMG, and spatio-temporal parameters acquired provided detailed insight into the relative contribution of different balancing strategies used to counteract perturbations and, more importantly, how this had changed after the training period.

### Balancing responses

This study shows that the coping strategies of (i) in-stance modulation of COP and GRF, and (ii) appropriate foot placement of the swinging leg act in a complementary manner throughout several steps following a perturbation and may, with appropriate training, be adequately modified to compensate for functional output that is missing due to stroke-related impairments.

The responses to LL-NP/L perturbations showed that, prior to the training, the individual post-stroke responded solely by an in-stance modulation of COP and GRF, primarily during the NP/L0-leg stance, and this lasted substantially longer than during unperturbed walking. This in-stance response was adequate only at the smaller perturbation amplitude and was comparable to the control response. The training brought about an important change that resulted in the individual post-stroke being able to place the paretic leg more laterally, something that is necessary to counteract perturbations of higher amplitude and which was not possible prior to the training.

On the other hand, the responses of the individual post-stroke to RR-P/R perturbations prior to the training displayed complete lack of an in-stance response on the paretic leg immediately after the commencement of perturbation, and this is frequently observed in chronic stroke [3]. The only coping strategy used consisted of a very fast first step of the left leg (NP/L1) which also entered the next stance substantially more laterally. It is interesting to note that during the second stance phase of the right leg (P/R1), the EMG recordings showed increased activity of GMED muscle; however, this activity was not accompanied by an adequate increase in GRFx, probably due to a co-contraction of hip adductors. After the training period, the left-leg (NP/L1) step was even faster and important changes were noticed in the in-stance responses of the right leg (P/R0, P/R1). Closer inspection of the GRFx after RR perturbation during two consecutive right-leg stance phases (from 0 to 50% of GC – P/R0, and from 100 to 150% of GC – P/R1) revealed that training brought about increase in the hip-strategy-based lateral force impulse that is important to contain the movement of COMx. This was more pronounced during the second right-leg stance phase (P/R1), where the magnitude of the GRFx force impulse was similar to the one present during the first right stance (P/R0) in the control response. This result suggests that training brought about changes that moved towards “normalization” of in-stance responses following RR-P/R perturbation in the individual post-stroke. Combining the in-stance response in subsequent P/R0 and P/R1 stances (Fig. 6) with the faster stepping response of NP/L1 and NP/L2 resulted in the individual post-stroke being able to successfully counteract higher-amplitude RR-P/R perturbations at the end of the training period that was not possible for him to deal with prior to the training.

Several studies have shown that, after a stroke, subjects display pronounced asymmetry in the time spent on non-paretic and paretic legs during walking [16, 17]. The stance time on the non-paretic side is substantially larger than on the paretic side, and this seemed to benefit the individual post-stroke who participated in this study when responding to the weaker (60 N) outward perturbations. After LL-NP/L perturbation, the longer stance time during the NP/L0-leg stance enabled in-stance modulation of COP and GRF by the non-paretic leg while, after RR-P/R perturbation, the shorter stance time in P/R0-leg stance enabled the non-paretic leg to be brought into a more laterally displaced stance as quickly as possible. However, prior to the training, particular deficiencies of the paretic leg in the individual post-stroke studied that were related to (i) a longer time needed to perform a swing and related inability to make a cross-step, and (ii) an inability to modulate COP and GRF under the paretic leg during a stance phase, had negative implications for his ability to counteract higher-amplitude perturbations. It was therefore important to develop balancing responses to outward-directed perturbations by progressively increasing the strength of perturbations, and this facilitated the observed changes in the use of the paretic right limb after the training.

### Self-induced perturbations in the sagittal plane

The results of this study show that the response to outward lateral perturbations also has important effects on movement in the sagittal plane. In our previous work, with a group of healthy subjects, we demonstrated the mechanisms underlying the deceleration of COMy following perturbations in outward directions [8] that the control participant in the current study also displayed. In the individual post-stroke, however, responding to an outward perturbation – regardless of whether it commenced on the non-paretic leg or paretic leg – induced an oscillation in COMy movement that may represent a considerable source of potential instability [4].

### Methodological considerations

We consider this study to be a pilot and, as such, a very important first step in exploring the feasibility and potential effectiveness of the novel proposed approach of robotic perturbation-based training of balance during treadmill walking post-stroke. Since only a single individual post-stroke was studied, the detailed assessment of balancing responses prior to and after the training allows us to state that the proposed methodology is potentially feasible and effective, but future studies involving more subjects are required to determine this.

After training, the individual post-stroke was able to handle perturbations under various conditions; however, to obtain meaningful comparisons, we have here reported only on performance at the single speed that was also used before training. Only one walking speed with two perturbation amplitudes was tested to avoid potential fatigue and lack of focus in the individual post-stroke. Further studies need to test various walking speeds, perturbation strengths, and timings for the onset of perturbations.

While the role of natural recovery cannot be completely ruled out, it is not very likely that the observed improvement in the abilities of the selected individual post-stroke to cope with rather strong outward perturbations could be attributed to spontaneous recovery since six months had elapsed since the stroke. Furthermore, since it has been suggested that traditional balance training approaches may not improve the capabilities of individuals post-stroke to cope with an unexpected loss of balance [10], and that such an improvement could be obtained through perturbation-based training based on the reduced number of falls [11], we may conclude that the observed improvement in the capabilities of the selected individual post-stroke to cope with perturbations is most probably a result of our intervention. Our robot-based approach to balance training may offer higher repeatability of individual pushes, in terms of both the amplitude and timing of a perturbation, than the perturbation-based balance approach of Mansfield et al. [11], who applied perturbing pushes/pulls manually, and such repeatability may be relevant for more efficient learning. This, however, needs to be investigated in future studies.

Another methodological limitation is that the perturbed-balance training took place on a treadmill where walking may be somewhat different to doing so in overground conditions. However, in a recent study, we demonstrated that the stepping responses of a group of healthy individuals to perturbations delivered by the BAR robot were substantially similar whether they occurred during treadmill or overground walking [18].

### Potential relevance

The results of this study may be very relevant for high-functioning, community-dwelling individuals post-stroke because training with BAR-TM may result in improvements in confidence that enable them to walk in busier places, such as shopping centers, where there is a higher probability of encountering balance-threatening situations. There was relatively modest improvement in the well-established clinical measures of balance and mobility for the individual post-stroke after the training. The FAC score was already maximal prior to the perturbed-balance training and scores in 6MWT, 10-MWT, TUG, and FSST were also well below the threshold that point to increased risk of falling at that time [13]. This confirms the notion that clinical scales may not detect the limited abilities of high-functioning individuals post-stroke to adequately respond to unexpected perturbations [2] and further underlines the need for a more objective, technological means for assessment of balancing responses during walking in realistic and fall-safe conditions [19]. To put the balancing abilities of the selected individual post-stroke after the training in the correct perspective, we should consider that in our previous studies [8, 12, 18], where the same assessment methodology was applied to healthy subjects, the maximum tolerable perturbation amplitude was around 15% of body weight. The selected individual post-stroke weighed 670 N, so amplitude of 90 N represents 13% of body weight and this indicates that, after the training, he had substantially improved ability to cope with LL-NP/L and RR-P/R outward perturbations.

## Conclusion

In this study, we implemented robot-based perturbed-balance training by applying forces to the pelvis of a walking individual post-stroke. The results have shown that this novel approach may be feasible and effective. We have provided a detailed insight into the mechanisms used in counteracting outward-directed perturbations through the assessment of kinematics, kinetics, EMG, and spatio-temporal parameters. The BAR-TM provided fall-free environment, enabling objective comparison of balancing abilities before and after therapeutic intervention. The positive outcome of this study warrants further studies investigating the application of robot-based perturbed-balance training in a group of individuals post-stroke.

## Additional file

Additional file 1: This video shows balancing responses of the individual post-stroke who participated in the study to the LL-NP/L and RR-P/R outward-directed perturbations delivered by the BAR-TM robot in the tenth of thirty consecutive training sessions. (MP4 19475 kb)
